# Military Medicine Publications: What has Happened in the Past Two Decades?

**DOI:** 10.2196/ijmr.2748

**Published:** 2014-05-28

**Authors:** Nirit Yavnai, Michael Huerta-Hartal, Francis Mimouni, Moshe Pinkert, David Dagan, Yitshak Kreiss

**Affiliations:** ^1^Israel Defense Forces Medical CorpsTel AvivIsrael; ^2^Israel Defense Forces Medical CorpsDepartment of Military MedicineHebrew UniversityJerusalemIsrael; ^3^Dana Dwek Children's Hospital, Tel Aviv Medical CenterSackler Faculty of MedicineTel Aviv UniversityTel AvivIsrael

**Keywords:** military medicine, publication types, trend

## Abstract

**Background:**

Military medical personnel, like all other physician specialists, face the challenge of keeping updated with developments in their field of expertise, in view of the great amount of new medical information published in the literature. The availability of the Internet has triggered tremendous changes in publication characteristics, and in some fields, the number of publications has increased substantially. The emergence of electronic open access journals and the improvement in Web search engines has triggered a significant change in the publication processes and in accessibility of information.

**Objective:**

The objective of this study was to characterize the temporal trends in the number and types of publications in military medicine in the medical literature.

**Methods:**

We searched all PubMed-registered publications from January 1, 1990 to December 31, 2010 using the keywords “military” or “army”. We used the publication tag in PubMed to identify and examine major publication types. The trends were tested using the Mann-Kendall test for trend.

**Results:**

Our search yielded 44,443 publications in military medicine during the evaluation period. Overall, the number of publications showed two distinct phases over time: (1) a moderate increase from 1990 to 2001 with a mean annual increase of 2.78% (*r* ^2^=.79, *P*<.002), and (2) a steeper mean annual increase of 11.20% (*r* ^2^=.96, *P*<.002) from 2002 to 2010. Most of the examined publication types showed a similar pattern. The proportion of high-quality-of-evidence publication types (randomized controlled trials, systematic reviews, and meta-analyses) increased from 2.91% to 8.43% of the overall military medicine publications with a mean annual incremental increase of 14.20%. These publication types demonstrated a similar dual phase pattern of increase (10.01%, *r* ^2^=.80, *P*<.002 for 1990-2001 and 20.66%, *r* ^2^=.88, *P*<.002 for 2002-2010).

**Conclusions:**

We conclude that over the past twenty years, scholarly work in the field of military medicine has shown a significant increase in volume, particularly among high quality publication types. However, practice guidelines remain rare, and meta-analyses are still limited in number.

## Introduction

### Scientific Journals on the Internet

At the end of the first decade of the twenty-first century, military medicine continues the evolving process of broadening its range, responsibilities, and resources, in combination with continued progress in developing unique skills and knowledge. Advances in health care, for instance, in the field of trauma care, often guide military medicine. Hence, military medical personnel, like other physician specialists, face the challenge of keeping updated with developments in their field of expertise. This is especially challenging in view of the great amount of new medical information regularly published in the literature [1,2]. Today, clinicians have access not only to PubMed, but also to many fast, comprehensive, Web-based data solutions, which can assist them in reaching current information directly related to their everyday practice [3]. Since 1950, the number of scholarly journals has increased rapidly, and today there are almost 30,000 peer-reviewed, indexed, English language journals, and several thousand additional journals published in languages other than English. In total, approximately 1.5 million articles are published annually [4]. The number of journals that address a specific field has increased over time and the Internet makes them easy to access [1,2]. The widespread availability of the Internet has triggered tremendous changes in publication characteristics [4-8]. Over the past two decades, the publishing process of scientific journals has undergone significant changes, due in part to the emergence of electronic open access journals, improvements in Web search engines, and the availability of specialized information services such as Clinical Evidence, UpToDate, and DynaMed [4,9].

### Military Medicine and Medical Knowledge

Military medicine is closely interwoven with a variety of medical specialties. Innovations in medical knowledge require implementation in military medicine, and medical challenges seen in the military environment may trigger further clinical research and development for general medicine.

### The PubMed Search Engine

Several search engines have been developed which allow easy access to relevant medical information. The National Library of Medicine offers PubMed as a free service that enables convenient searches of medical publications. PubMed is more than a search engine; it is also an extremely large, free, and highly reputable database of the biomedical and health care literature. PubMed uses a defined system of categorization of medical publications. Among these publication types, one can find clinical trials, reviews, editorials, meta-analyses, randomized controlled trials (RCTs), and practice guidelines, as well as additional publication types.

### The Aim of the Study

The aim of this study was to characterize the overall and publication type-specific temporal trends of scientific publications in military medicine over the last two decades.

## Methods

### PubMed Search Engine and Keywords

We used the PubMed search engine on June 5, 2012 to examine all of the articles indexed from January 1, 1990, through December 31, 2010, searching for publications in the field of military medicine. A military medicine publication was defined as an article published in a military medicine journal, or, if the subjects/participants were soldiers/veterans or army personnel, or, if the author affiliation was a military hospital or army medical institute. Our search algorithm was based on several exploratory terms including "military", "army", "combat", "war", "soldier", "battle", "terror", and "weapon". These terms were highly sensitive, but nonspecific, and returned articles outside of the intended field of military medicine. We attempted several keyword combinations, which we validated by examining the abstracts of the first ten articles in each type for a single publication year (2010). Upon completion of this process, the final keyword terms selected were "military OR army". An alternative search strategy we considered, involved using medical subject headings (MeSH) terms. MeSH is the National Library of Medicine's controlled vocabulary thesaurus, which consists of sets of terms in a hierarchical structure that permits searching at various levels of specificity to select specific fields of medicine [10]. However, using a MeSH-based search strategy would not have significantly increased the number of publications returned. Our search was limited to human subjects and to English language publications, and we sorted our results by PubMed publication type (ie, clinical trials, reviews, systematic reviews, meta-analyses, editorials, letters, case reports, practice guidelines, and historic articles). The PubMed engine has previously been found to accurately determine the publication type 100% of the time [1,2,6,7]. In order to verify and validate the accuracy of the publication type in the present study, we drew a random sample of 10 articles from 5 randomly selected years for manual evaluation. In all cases, the publication type was found to be accurate, but there was some overlap. For example, RCTs may also be listed as clinical trials, and some systematic reviews are also classified as meta-analyses.

### Statistical Analysis

The statistical analysis was performed using the WinPepi software (version 11.25, October 2012) [11]. We first conducted an empirical evaluation of the overall distribution of values over time. We hypothesized that the incremental trends over time in publication characteristics would not remain constant throughout the study period, and that the later years would reflect a steeper increase than what was represented in the earlier years. The values of each time period were tested for the trend using the Mann-Kendall test. This is a nonparametric test of monotonic trend over time [12], for which two tailed *P*-values are reported, since our original hypothesis did not assume an upward or downward direction over time. Upon identifying the optimal cut-off year, we calculated the mean annual incremental change in publication volume for the overall publication dataset and for the subset of high quality publications. We considered RCTs, systematic reviews, and meta-analyses, generally considered to reside at the uppermost levels of the pyramid of evidence, to be high quality publications [9,13]. Additionally, we fitted independent linear regression models to the earlier (1990-2001) and later (2002-2010) time periods. The above procedures were carried out for the overall dataset. We then performed a subanalysis after stratifying the data by publication type. For each linear regression model, we reported the coefficient of determination (*r* ^2^), which provides a measure of the goodness-of-fit of the regression line to the observed data [14].

## Results

### The Shift in the Character of the Trend

**Figure 1:**
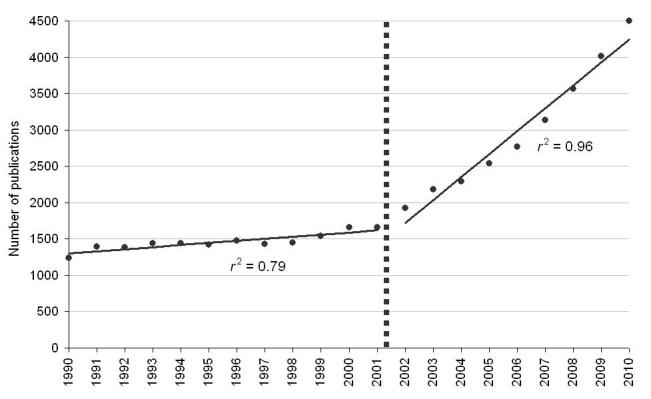
Total number of publications in military medicine by year of publication.

During the 21 year period, PubMed reported 44,443 publications related to military medicine, with a mean of 2116.3 publications per year (SD 958.6). We discerned two distinct phases, with a shift in the character of the trends over time up to and after 2001. From 1990-2001, we identified a linear progression with a moderate slope, indicating a mean annual increase of 2.78% in the publication volume, increasing from 1238 in 1990 to 1658 in 2001. From 2002 to 2010, there was a substantial change in the trend, with an increased slope (Figure 1 shows this slope) from 1929 publications in 2002, to 4497 in 2010, and a mean annual increase of 11.20% in the publication volume. The coefficient of determination (*r* ^2^) was .79 for the initial segment (Mann-Kendall test for trend *P*<.002), and 0.96 for the subsequent steeper segment (*P*<.002).

### The Subset of High Quality Publications

**Figure 2:**
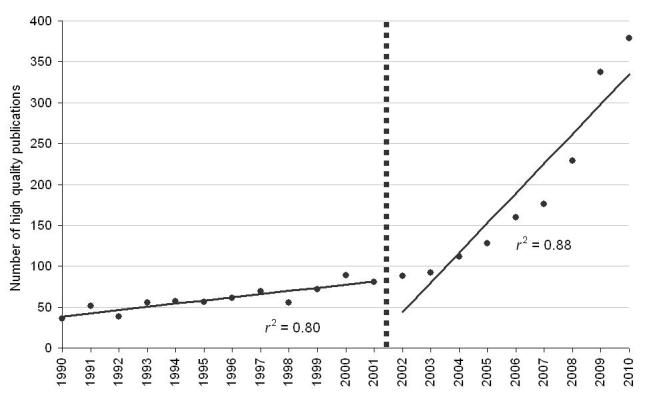
Number of high quality publications by year of publication.

The subset of high quality publications (RCT, systematic review, and meta-analyses) represented 5.45% (2421/44,443) of all the publications during the overall evaluation period, with a mean of 4.86, SD 1.48% annually (Figure 2 shows the number of publications). There was an increase in this proportion over time from 2.91% in 1990 to 8.43% in 2010. The mean overall annual increment in high quality publication types was 14.20%. This increase showed two phases as well, one for 1990-2001, with a mean annual increment of 10.01% (*r* ^2^=.80, *P*<.002); and a second phase for 2002-2010, with a mean annual increment of 20.66% (*r* ^2^=.88, *P*<.002).

Table 1 presents the mean number of annual publications, mean annual incremental change and *r* ^2^ for the two time phases (1990-2001 and 2002-2010), and the overall study period, stratified by publication type.

Most of the publication types showed a moderate mean annual incremental change in the early phase, and a steep increase in the later phase. The most common publication type in the lower quality category was the case report (15.01%, 6671/44,443). Case reports were published during the early phase at a gradually decreasing annual rate of 2.02%. In the later phase, however, this trend was reversed, and the publication of this article type proceeded at an annual rate of 10.89%. During the same time periods, the annual rate of increase for clinical trials nearly doubled from 7.05% in the early phase to 13.02% in the later phase. The trends for editorials and practice guidelines (1.02% and 0.06% respectively) were much less stable, owing mostly to the small absolute number of these article types.

Editorials and case reports showed a linear decrease in publication volume during the early phase, but still increased during the later phase.

**Table 1:** The mean number of annual publications, mean annual incremental change and *r* ^2^ for the two time phases (1990-2001 and 2002-2010), and the overall study period, stratified by publication type.

Publication type			All years, 1990-2010			Phase 1, 1990-2001			Phase 2, 2002-2010		
			n (%)	Mean annual publications (SD)	Mean annual % incremental change	Mean annual % incremental change	*r* ^2^	*P* value^a^	Mean annual % incremental change	*r* ^2^	*P* value^a^
All articles			44,443 (100)	2116.3 (958.6)	6.83	2.78	.79	<.002	11.20	.96	<.002
**Article types**											
	**High quality**		2421 (5.4)	115.3 (94.4)	14.20	10.01	.80	<.002	20.66	.88	<.002
		RCT	1472 (3.3)	70.1 (38.0)	10.29	6.87	.54	<.02	16.95	.91	<.002
		Systematic review	736 (1.7)	35.1 (41.8)	29.90	30.70	.69	<.002	27.18	.84	<.002
		Meta- analyses	213 (0.5)	10.1 (16.6)	67.78	75.0	.52	<.02	65.0	.75	<.02
	**Other selected types**		17,960 (40.4)	855.2 (307.9)	5.33	1.42	.39	<.05	10.54	.99	<.002
		Case reports	6671 (15.0)	317.7 (113.9)	4.58	-2.02	.51	<.02	10.89	.98	<.002
		Reviews	4507 (10.1)	214.6 (90.8)	6.86	3.99	.63	<.002	10.20	.96	<.002
		Clinical trials	2718 (6.1)	129.4 (59.8)	9.23	7.05	.73	<.02	13.02	.96	<.002
		Historical articles	2352 (5.3)	112.0 (37.3)	8.97	11.83	.59	<.02	8.03	.54	<.05
		Letters	1234 (2.8)	58.8 (11.9)	1.91	12.59	.54	<.1	9.71	.46	<.1
		Editorials	452 (1.0)	21.5 (16.1)	15.08	-2.00	.58	<.02	37.32	.71	<.05
		Practice guidelines	26 (<0.1)	1.2 (1.2)	-4.55	-25.0	.03	>.2	31.25	.04	>.2

^a^Mann Kendall test for the trend

## Discussion

### Increase in Military Medicine Publications

The aim of this study was to characterize the overall and publication type-specific trends of scientific publications in military medicine over the last two decades. Over the study period, the number of publications related to the field of military medicine increased steadily. The rate of increase over time was not constant, as can clearly be seen from the figures. The slope indicating the mean annual incremental change increased substantially after 2001. This period coincides with the start of the global war on terror, and we speculate that the increased number of publications subsequent to that point in time is a derivative of this development. Alternatively, it is possible that this change is due to the temporal trends across disciplines and different publication types within PubMed. The observed pattern over time held true for most article types. The proportion of high quality research publications (RCT, systematic review, and meta-analysis) increased significantly over time. We interpret this as an indication of the increasing overall quality of the research conducted in the field of military medicine. Meta-analyses were hardly known in the 1990s [1], and very few were published annually in the field of military medicine during the first years of our study period. An increase in the annual number of published meta-analyses to several dozen per year explains the high annual incremental change, although in absolute terms, there is clearly more room for this article type in the future.

The results of the regression analysis for the high quality publication types showed an increase in *r* ^2^ values, from a range of 0.52 to 0.69 in the first phase, to a range of 0.75 to 0.91 in the second phase. A similar trend was observed for other publication types as well, increasing from a range of 0.51 to 0.73 in the first phase to 0.46 to 0.98 in the second phase. A notable exception to this pattern was practice guideline publications, which demonstrated extremely low *r* ^2^ values throughout the entire study period. This was most likely due to the very small number of guidelines published (26 in total over the 21 year period). A likely interpretation of this finding is that military treatment guidelines are often based upon general clinical guidelines published in the medical literature, so that military-specific practice guidelines are not necessary. Furthermore, it is possible that detailed guidelines developed especially by military medical personnel for operational purposes are classified, and thus are not published in the scientific literature.

### Limitations

Our study has several limitations. It is possible that our keyword search terms underestimated the overall number of military medical publications. Furthermore, the publication typing and tagging that is offered by PubMed may not be entirely accurate, with potential overlap between publication types. A misclassification error in publication type is also possible, for example, systematic reviews may not always be accurately identified as such by PubMed, since search filters do not always differentiate between the systematic reviews and meta-analyses and guidelines. (There is one such filter that is available at PubMed under "Clinical Queries", see the PubMed website). However, a random sample of retrieved articles examined manually showed an excellent degree of agreement with PubMed tags, and any overlap was observed mainly within the high quality publication types, specifically between RCTs and clinical trials, and between reviews, systematic reviews, and meta-analyses.

Our search strategy included publications whose authors carried a military affiliation. This strategy has yet to be validated, and it is possible that our data included articles published by military scholars, even if the subject matter was not directly associated with military medicine. Alternatively, our strategy may have included publications by scholars affiliated with military hospitals, but whose research was not necessarily military in nature. If this were true, it would be expected to increase the false-positive rate of our sample. Future research in this topic should consider alternative search strategies in order to enable a comparison between different methods.

### Conclusions

In conclusion, over the past twenty years the field of military medicine has witnessed a significant increase in the publication of scholarly articles under various publication types, especially those considered to be of high quality. However, practice guidelines remain rare, and meta-analyses are still limited in number. We speculate that the increasing accessibility and availability of electronic resources to readers and authors will generate additional changes in publication trends in this field in the future.

## Abbreviations

MeSH: medical subject headings
RCT: random controlled trials
